# A Rabbit Corneal Endothelial Dysfunction Model Using Endothelial-Mesenchymal Transformed Cells

**DOI:** 10.1038/s41598-018-35110-2

**Published:** 2018-11-15

**Authors:** Kazuya Yamashita, Shin Hatou, Emi Inagaki, Kazunari Higa, Kazuo Tsubota, Shigeto Shimmura

**Affiliations:** 1 Department of Ophthalmology, Keio University School of Medicine, Tokyo, Japan; 2 Department of Ophthalmology, Tokyo Dental College Ichikawa General Hospital, Ichikawa, Japan

## Abstract

Unlike humans, rabbit corneal endothelial wounds are known to spontaneously heal. The current study was aimed to develop a new rabbit bullous keratopathy model using corneal endothelial cells that were induced to undergo endothelial-mesenchymal transformation (EMT). EMT was induced in rabbit corneal endothelial cells (RCECs) by culturing with TGFβ and basic FGF Supplemented Medium. The corneal endothelia in recipient rabbits were mechanically scraped from the corneal endothelial surface inside an 8 mm mark. Then, a suspension of EMT-induced RCECs (EMT-RCECs) was injected into the anterior chamber. Eyes injected with freshly isolated RCECs (Fresh RCECs group) and eyes that were scraped without injection of cells (Scrape group) were used as controls. Immediately following operation, subepithelial and stromal edema was observed with increased central corneal thickness and corneal opacity in all groups. In the EMT-RCECs group, bullous keratopathy persisted for 42 days up to the end of the study. In the Fresh-RCECs and Scrape groups, corneal transparency and thickness recovered by 7 days after treatment and was maintained up to 42 days. The activated fibroblast marker, α-SMA, was observed spanning from corneal endothelium to corneal stroma in the EMT-RCECs group. Interestingly, α-SMA was upregulated in the Scrape-group as well. In all groups, there was no damage to other intraocular structures, and intraocular pressure was normal throughout the observation period. Transplanting a fresh donor cornea effectively treated corneal edema due to bullous keratopathy. This model is a promising tool for pre-clinical trials in the development of new therapies against corneal endothelial dysfunction.

## Introduction

Corneal transparency is maintained by the corneal endothelium through the barrier function of tight junctions and by Na^+^/K^+^-ATPase pumps located along the lateral cell walls^1^. Corneal endothelial dysfunction causes irreversible loss of corneal transparency, and is a major cause of visual impairment and corneal blindness. Although efforts to develop pharmaceutical agents to treat corneal endothelial dysfunction demonstrate promising results^2–5^, corneal transplantation is still the standard treatment of choice. In order to develop new therapies for corneal endothelial disease, an animal model that resembles human corneal dysfunction is required. Several investigators have studied corneal endothelial dysfunction models using cryo-injury^6–8^, ultrasonic emulsification^9^, Nd:YAG laser^10^, chemical injury (intracameral injection of povidone–iodine^11^, benzalkonium bromide^12^ and NaOH^13^), mechanical scraping of corneal endothelium^14^ or stripping of Descemet membrane with corneal endothelium^15–18^. Animal species used in these models include the rabbit^6–8,10,11,13–15^, mouse^12^, rat^19^, cat^7^, pig^20–22^ and monkey^9,23,24^. However, the reliability and stability of small animal models are less than ideal, and monkeys are very expensive. The rabbit model is the most popular because of low cost and a similar corneal diameter compared to humans^25^. Unfortunately, unlike humans, rabbit corneal endothelial cells (RCECs) proliferate *in vivo* and wounds tend to heal spontaneously^7^. In addition, previous animal models have limitations such as increased intraocular pressure (IOP) or glaucoma, anterior chamber inflammation and tissue damage. Also, models that involve acute damage to the central cornea to create a defect in the corneal endothelium do not reflect the pathology of chronic bullous keratopathy in humans. A state in which chronic corneal edema occurs due to functional deterioration of diseased (or aged) corneal endothelial cells is the pathophysiology of bullous keratopathy in humans, and an appropriate animal model that mimics this state is required.

Recently, it has been shown that fibroblastic cells play a role in the healing of corneal wounds^26,27^. Fibroblastic cells found in the damaged corneal stroma are most likely derived from keratocytes residing in the stroma. On the other hand, corneal endothelial cells in rabbits reportedly also showed fibroblastic transformation during the wound healing process^28^, and IL-1β–mediated FGF-2 produced after injury reportedly alters corneal endothelial cell morphology and the actin cytoskeleton^29^. Basic fibroblast growth factor (bFGF) is the direct mediator for corneal endothelium modulation by cultured corneal endothelial cells^30,31^. It is possible that the rabbit corneal endothelial cells may transform and proliferate to a fibroblastic cell type and participate in fibroblastic role in the corneal stroma. This speculation is especially interesting when considering that corneal endothelial cells are derived from the neural crest during embryonic development, just as are the stromal keratocytes^27,32^. We therefore hypothesized that corneal endothelial dysfunction in humans can be imitated in a rabbit model by using corneal endothelium that were induced to undergo endothelial- mesenchymal transformation (EMT) in culture with TGFβ and basic FGF Supplemented Medium. We show how EMT-induced RCECs (EMT-RCECs) maintains corneal endothelial dysfunction for up to 42 days, which can then be treat successfully with penetrating keratoplasty.

## Results

### Cultivated RCECs with SEMTM undergo EMT *in vitro*

**Figure 1:**
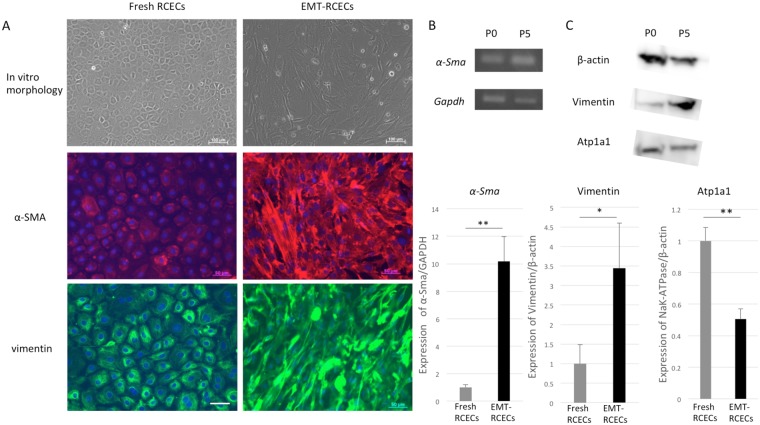
Endothelial-mesenchymal transformation (EMT) of RCECs using specific endothelial-mesenchymal transformation medium (SEMTM). (**A**) Immunohistochemistry of the fibroblastic markers α-SMA and vimentin were characteristic of fibroblasts in EMT-RCECs compared to the hexogonal morphology of Fresh RCECs (Scale bar upper panel = 100 µm, Scale bar middle and bottom panel = 50 µm). (**B**) RT-PCR showed that both Fresh RCECs and EMT-RCECs expressed *α-Sma*. Original gel is shown in Supplemental Fig. S2. However, qRT-PCR in the lower panel shows *α-Sma* was significantly upregulated in EMT-RCECs. (**C**) Western blots showed that Fresh RCECs and EMT-RCECs expressed Vimentin, Atp1a1, a marker of differentiated endothelial cells, and b-actin. Original gel and blots are shown in Supplemental Figs S3, 4. Semiquantitative analysis shows significant upregulation of the protein levels of Vimentin (p = 0.048) and downregulation of the protein level of Atp1a1 (p = 0.0043). Data expressed as mean ± SD of three replicate experiments. Student’s *t* test (*p < 0.05, **p < 0.01). (n = 3). RCECs: rabbit corneal endothelial cells, α-SMA: smooth muscle α-actin. Atp1a1: Na,K‐ATPase α‐subunit.

Fresh RCECs showed a polygonal morphology characteristic of endothelial cells. These cells were cultured and passaged with endothelial-mesenchymal transformation medium (SEMTM), and the cell morphology changed to a fibroblastic, spindle morphology. To assess the effect of RCECs cultured with TGFβ and basic FGF Supplemented Medium, the histological phenotype of RCECs was evaluated by immunofluorescence for α-SMA and vimentin as a marker of fibroblastic change. α-SMA and vimentin were evident in EMT-RCECs, whereas they were less observed in the fresh corneal endothelium (Fig. 1A). The fibroblastic marker *α-Sma* (p = 0.0079) was significantly upregulated after passaging RCECs (passage 5, EMT-RCECs) as shown by qRT-PCR (Fig. 1B). Western blots of vimentin, corneal endothelial markers Atp1a1 and β-actin were expressed. However, EMT-RCECs expressed significantly higher protein levels of vimentin (p = 0.048) and significantly lower levels of Atp1a1 (p = 0.0043) compared to Fresh RCECs (Fig. 1C). These findings indicated that RCECs have been induced to undergo EMT by cultured with SEMTM.

### Injected EMT-RCECs show fibroblast-like cell morphology after engraftment on Descemet’s membrane

**Figure 2:**
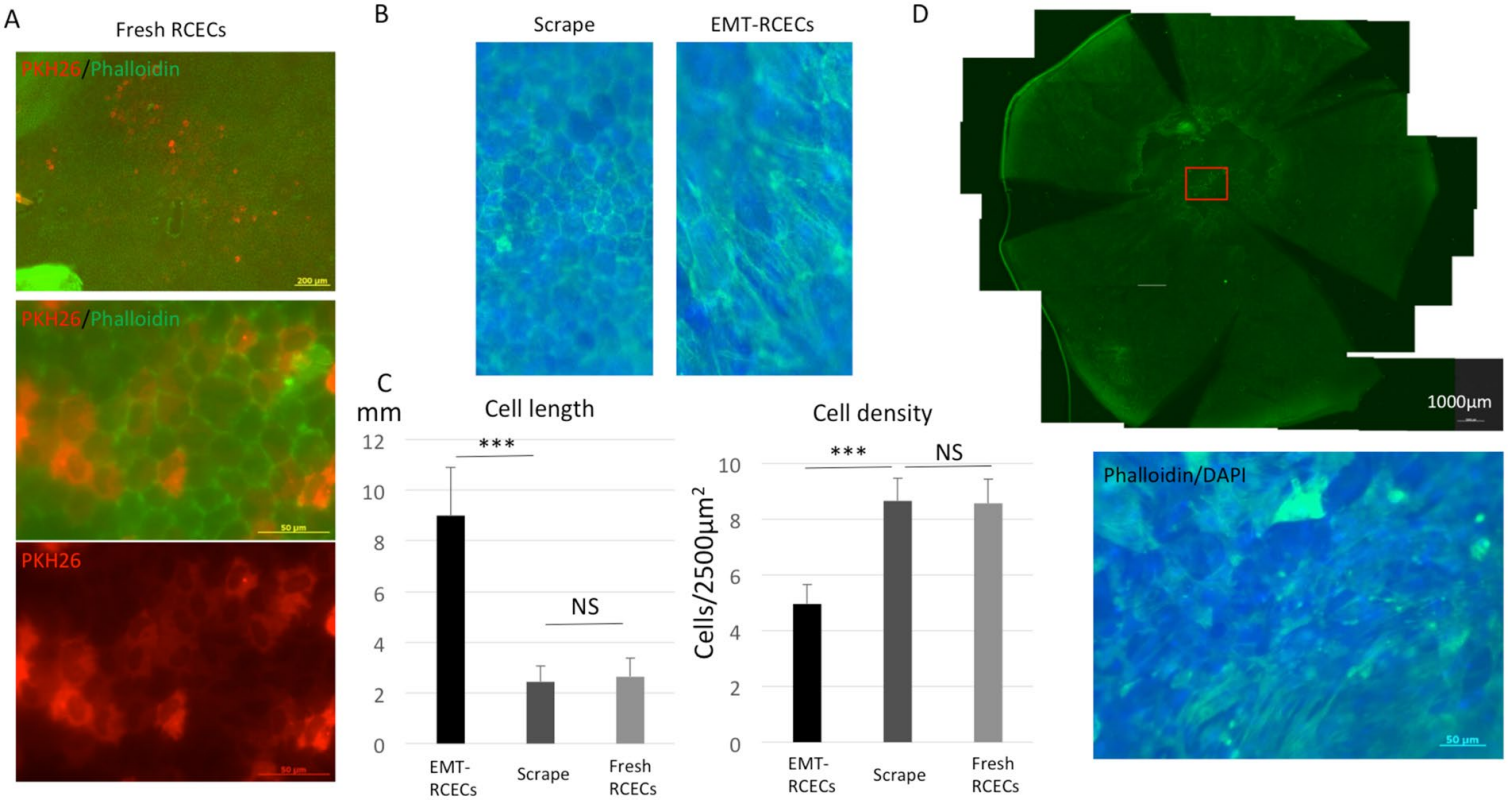
Rabbit corneal endothelial scrape model injected with EMT-RCECs and Fresh RCECs (**A**) Two weeks after injection of Fresh RCECs, donor-derived PKH26 positive cells were engrafted on Descemet’s membrane. Phalloidin staining showed a polygonal structure characteristic of normal endothelial cells. (**B**) Host corneal endothelial cells proliferated and/ or migrated in the Scrape-group after 6weeks to recover a single layer of endothelial cells with characteristic polygonal morphology shown by phalloidin staining. On the other hand, injected EMT-RCECs showed fibroblast-like morphology. (**C**) Average cell length of injected EMT-RCECs were significantly longer than scraped RCECs (p = 6.2 × 10^−16^) or injected Fresh RCECs (p = 6.4 × 10^−20^). The cell length was not significant different between the Scrape-group and Fresh RCECs injected group (p = 0.16). Cell density after EMT-RCECs injection was significantly lower than the Scrape-group (p = 1.6 × 10^−20^) or Fresh RCECs group (p = 2.1 × 10^−19^). The cell density was not significantly different between the Scrape-group and Fresh RCECs group (p = 0.37). Multiple *t* test with Bonferroni correction after ANOVA. n = 25. ***P < 0.001. NS: not significant. (**D**) Six weeks after EMT-RCECs injection, phalloidin staining showed fibroblast-like irregular cells in the central cornea on the Descemet’s membrane. (Scale bar upper panel = 1000 µm, bottom panel = 50 µm).

We performed immunohistochemistry for laminin in scraped and intact corneas to examine the amount of damage done to Descemet’s membrane using the scraping technique. A layer of laminin remains intact on the Descemet’s membrane surface following cell scraping. (Supplementary Fig. S1). In the rabbit corneal endothelial dysfunction model, Fresh RCECs stained with PKH26 were engrafted on Descemet’s membrane. Phalloidin staining showed polygonal morphology characteristic of RCECs (Fig. 2A). Scrape-group also showed polygonal corneal endothelial cells that were a result of normal wound healing. On the other hand, EMT-RCECs showed a fibroblast-like cell morphology (Fig. 2B). The average cell length of injected EMT-RCECs was significantly longer than the Scrape-group (p = 6.2 × 10^−16^) or Fresh RCECs (p = 6.4 × 10^−20^). Cell length was not significant different between Scrape-group and Fresh RCECs (p = 0.16). The average cell density of injected EMT-RCECs was significantly lower than the Scrape-group (p = 1.6 × 10^−20^) and Fresh RCECs (2.1 × 10^−19^). Cell density was not significant different between Scrape-group and Fresh RCECs (p = 0.37) (Fig. 2C). Six weeks after EMT-RCECs were injected in the rabbit corneal endothelial dysfunction model, flat-mount examination of the posterior side of the corneal tissue stained phalloidin showed fibroblast-like cells with irregular morphology on the Descemet’s membrane (Fig. 2D).

### Long-standing rabbit bullous keratopathy model

**Figure 3:**
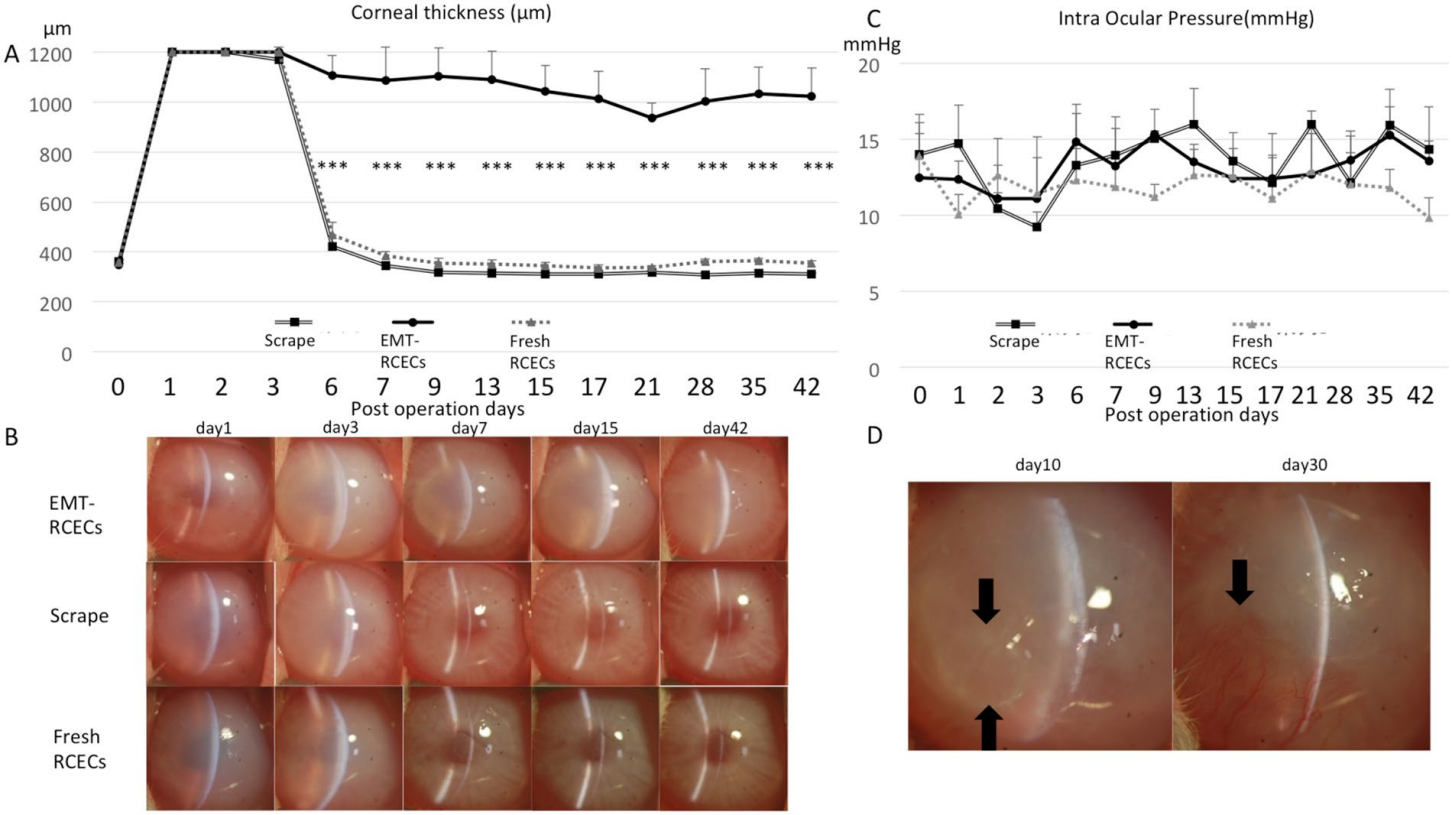
Functional assessment of *in vivo* transplantation. (**A**) Change in corneal thickness after transplantation shows significant increase in thickness due to edema in the EMT-RCECs group. Corneal thickness 42 days postoperatively in the EMT-RCECs group was higher than Scrape-group (p = 2.0 × 10^−5^) and also higher than the Fresh RCECs group (p = 2.9 × 10^−5^) (***p < 0.001). (**B**) Anterior segment photographs of rabbit corneas 1, 3, 7, 15, 42 days after transplantation. Eyes transplanted with EMT-RCECs showed severe edema (upper panel), while Scrape-group and Fresh RCECs group recovered transparency after 7 days (middle and lower panel). (**C**) IOP was similar in all groups indicating that the difference in corneal thickness was not due to differences in IOP. (Scrape-group: black squares *n* = 4), (EMT RCECs: black circles *n* = 4), (Fresh RCECs: gray triangles *n* = 4). (**D**) Anterior segment photographs of rabbit corneas 10 (left panel) and 30 (right panel) days after transplantation with EMT-RCECs. Bullae formation is observed in day 10 (arrow), sub-epithelial changes such as neovascularization after day 30 (arrow). Abbreviation IOP: intraocular pressure.

To assess whether EMT-RCECs can be used as a tool for long-standing, non-healing corneal endothelial dysfunction, we transplanted cell suspensions in a rabbit corneal endothelium dysfunction model. Figure 3A showed corneal thickness change in the three experimental groups. During 42 days after transplantation, corneal thickness of EMT-RCECs increased over 1000 µm (at 42 days 1023.13 ± 115.73 µm) and revealed severe corneal cloudiness consistent with bullous keratopathy, whereas Scrape-group and rabbits injected with Fresh RCECs maintained significantly lower corneal thickness than EMT-RCECs by 7 days following surgery up to 42 days (Scrape-group 310.88 ± 3.20 µm, Fresh RCECs group 354.81 ± 8.63 µm). Figure 3B shows slit lamp photographs of rabbit eyes 1, 3, 7, 15 and 42 days after transplantation. Corneas of EMT-RCECs group suffered severe corneal edema and thickened stroma throughout the 42 day observation period (Fig. 3B: top panel), whereas corneas of Scrape-group and Fresh RCECs group were less edematous, and maintained transparency after 7 days (Fig. 3B: middle and bottom panel). Figure 3C shows the changes in intra ocular pressure (IOP) during the observation period. IOP was maintained within the normal range of 10–20 mmHg in all groups. There was no significant difference between groups, indicating that the difference in corneal thickness was due to EMT-RCECs pump function, but not IOP. Figure 3D shows bullae formation is observed in this model, as well as sub-epithelial changes such as neovascularization after an extended period of time. These findings indicate that EMT-RCECs after transplantation can cause bullous keratopathy in rabbits over the long term.

### Immunohistochemistry and histopathological examination of EMT-RCECs-induced corneal endothelial dysfunction

**Figure 4:**
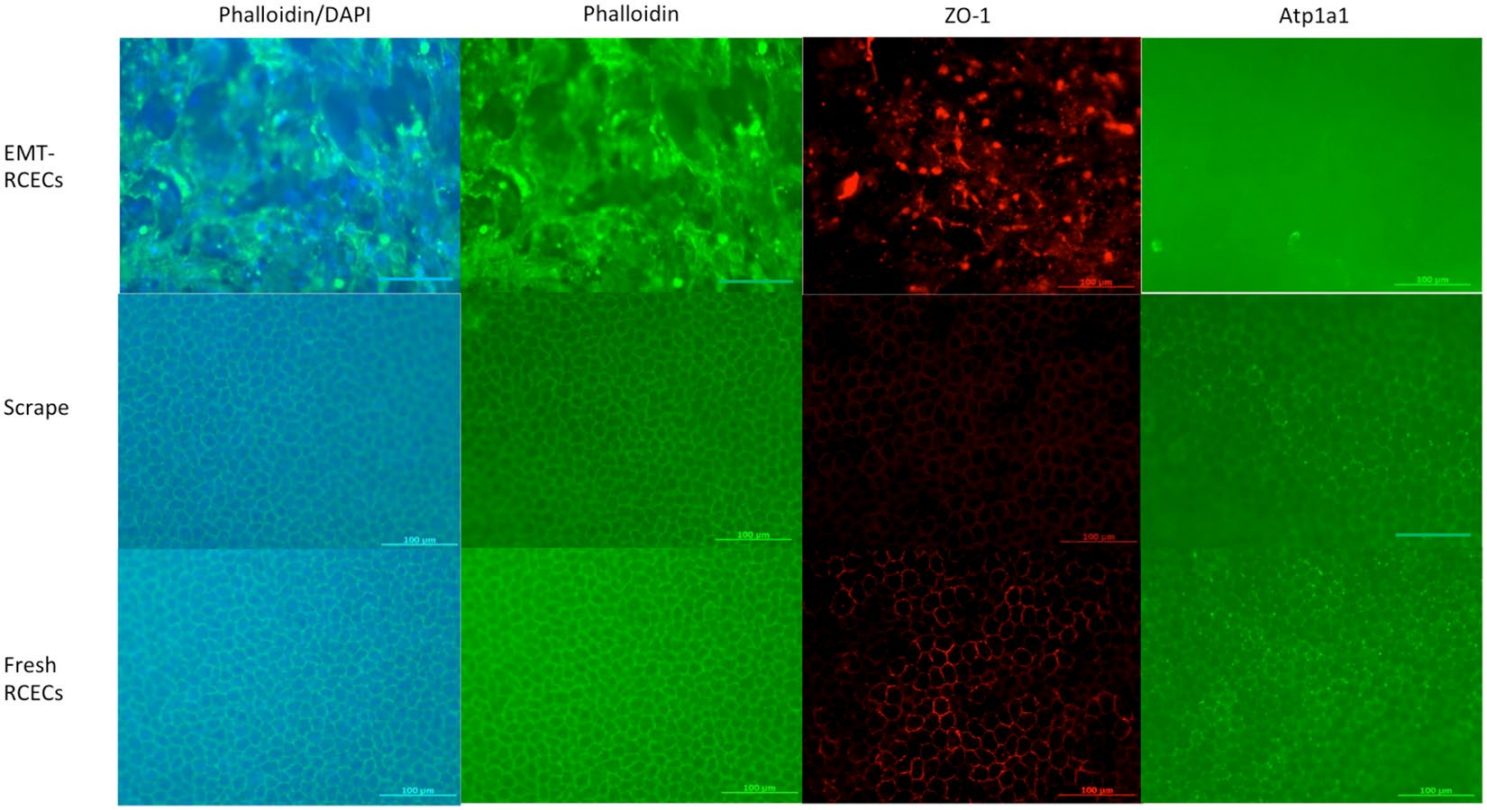
Immunohistochemistry of ZO-1, Na^+^/K^+^-ATPase and phalloidin. Histological examination of corneal tissue taken from the rabbit eye 6 weeks after transplantation (Top panel: EMT-RCECs group, middle panel: Scrape-group, bottom panel: Fresh RCECs group). Monolayer of hexagonal cells were observed in the Fresh RCECs group and Scrape-group. Irregular fibroblastic cells were observed in the EMT-RCECs group. The expression of ZO-1 and Na^+^/K^+^-ATPase was evident in Scrape-group and Fresh RCECs group, but it was not observed in the EMT-RCECs group. Scale bar = 100 μm.

Histological examination of corneal tissue 6 weeks after transplantation shows phalloidin-positive polygonal cells in the Fresh RCECs and Scrape group, while irregular fibroblastic cells were observed in the EMT-RCECs group. The main function of corneal endothelial cells is to prevent corneal edema by tight junctions formed by zonular occludens-1 (ZO-1) and Na, K-ATPase pump function. Immunohistochemistry of ZO-1 (red) and Na, K-ATPase (green) in EMT-RCECs outlined fibroblastic cell morphology, and were less expressed compared to Fresh RCECs group and Scrape-group. Immunohistochemistry revealed the localization of Na, K-ATPase and ZO-1 in cell-cell junctions, suggesting the presence of tight junctions in Scrape-group and Fresh RCECs group (Fig. 4).

**Figure 5:**
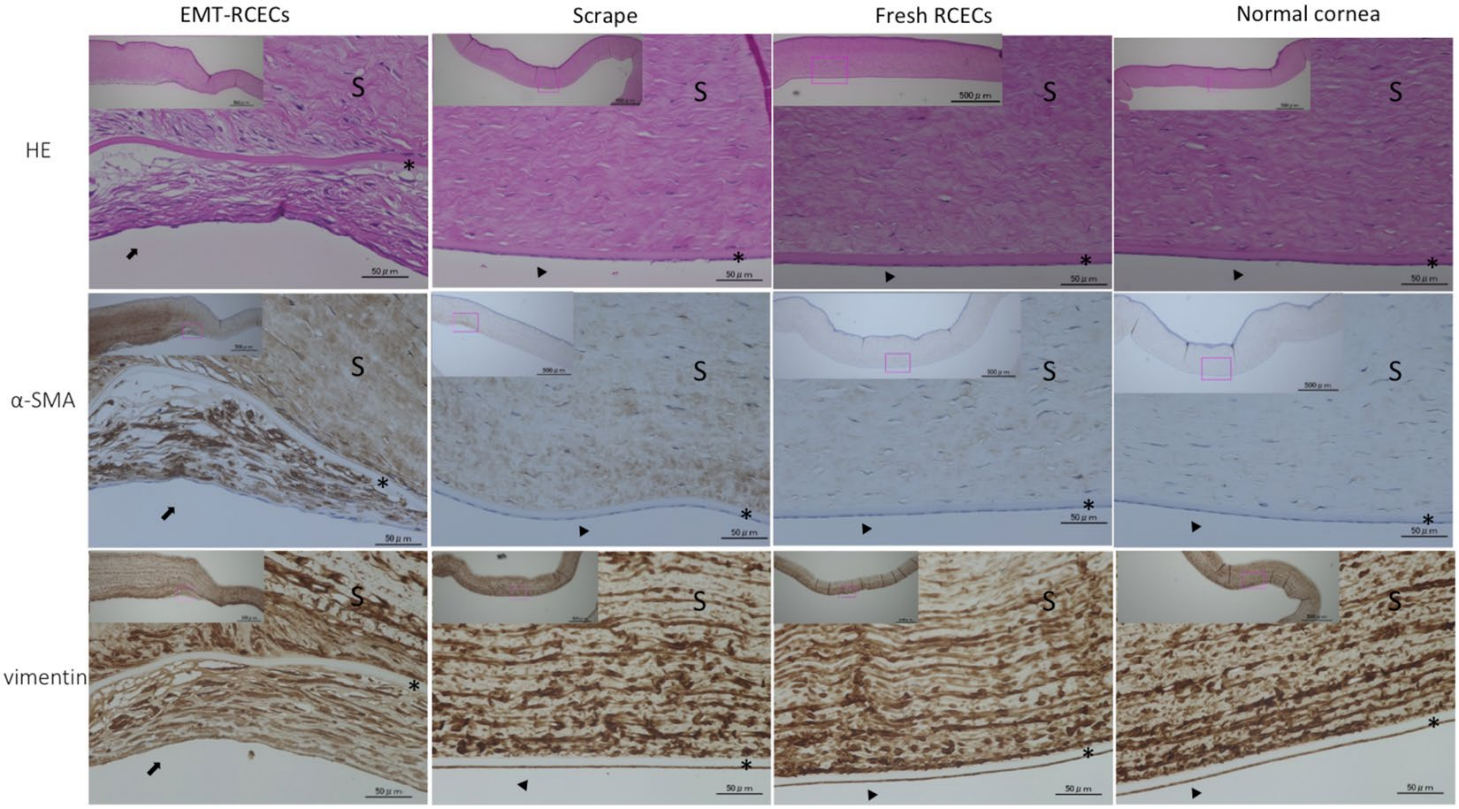
Histological examination by Hematoxylin and Eosin (HE), smooth muscle α-actin (α-SMA) and vimentin staining. Histological analysis of EMT-RCECs group, Scrape-group, Fresh RCECs group and untreated normal corneas (top panel: Hematoxylin and Eosin (HE) staining; middle panel: smooth muscle α-actin (α-SMA) staining, bottom panel: vimentin staining at 6 weeks after injection). Injected EMT-RCECs were engrafted on Descemet’s membrane with fibroblast-like morphology. α-SMA staining was also observed in the stroma of the Scrape-group, while Fresh RCECs group and normal RCECs only showed weak staining. Vimentin staining was similar in all groups. Scale bars = The upper left panel 500 μm, center panel 50 μm. *Descemet’s membrane was intact. Transplanted EMT-RCECs formed a confluent layer on the Descemet’s membrane. (arrow). Corneal endothelial cells formed monolayers (arrow head). Abbreviation S: Corneal stroma, *Descemet’s membrane.

Hematoxylin and Eosin (HE) at 42 days showed multilayered fibroblast-like corneal endothelial cells attached tightly on Descemet’s membrane in the EMT-RCECs group. In Fresh RCECs and Scrape-groups, a monolayer of corneal endothelial cells were observed on the Descemet’s membrane. The activated fibroblast marker α-SMA was observed spanning from the corneal endothelium to corneal stroma in the EMT-RCECs group. Interestingly, α-SMA staining was also expressed in the stroma of the Scrape-group, followed by the Fresh RCECs group. Vimentin was expressed by most stromal cells in all groups (Fig. 5).

### Successful treatment of long-standing bullous keratopathy by penetrating keratoplasty (PKP)

**Figure 6:**
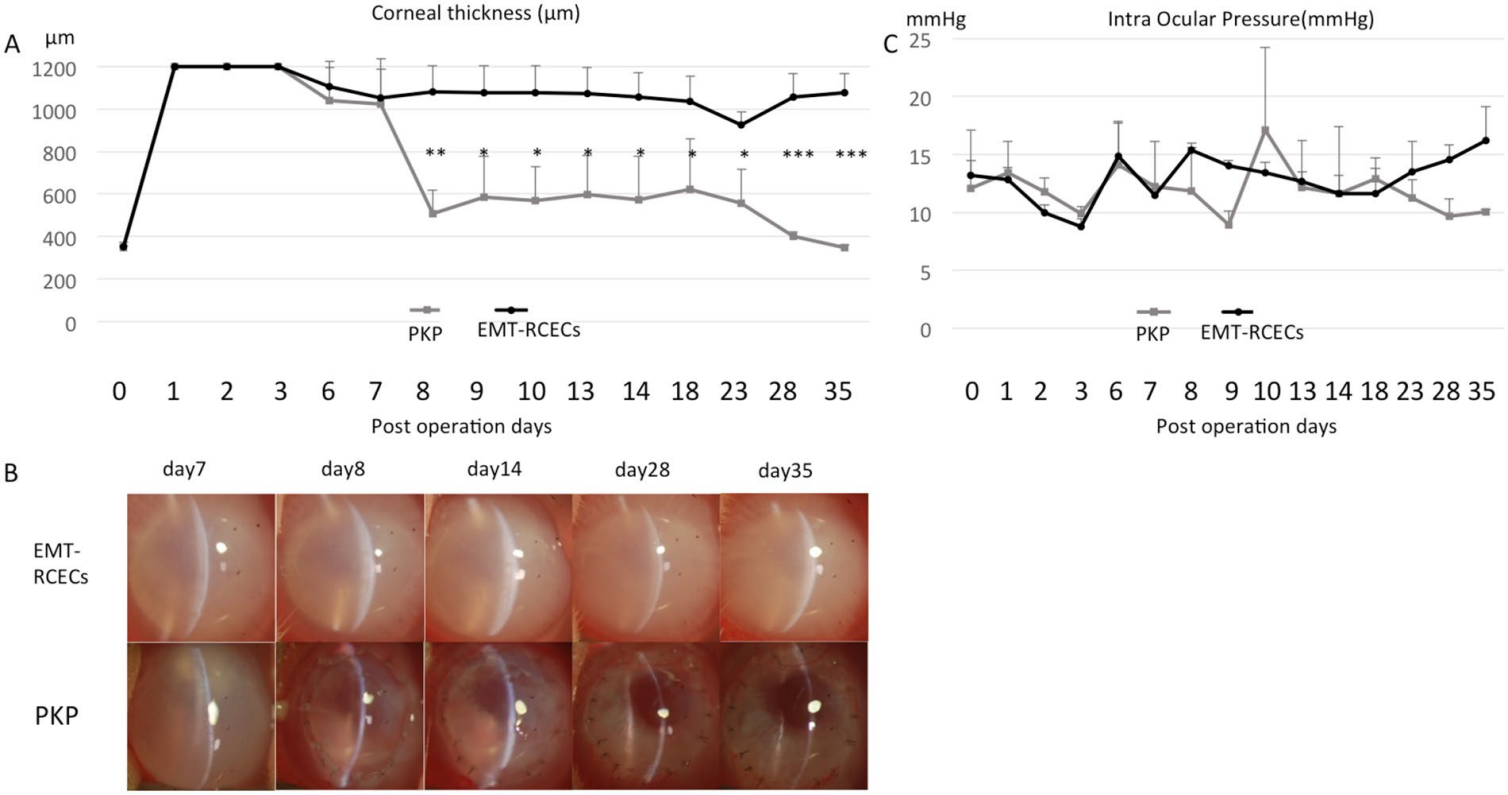
Functional assessment after penetrating keratoplasty (PKP) performed 7 days after EMT-RCECs injection (**A**) Corneal thickness after penetrating keratoplasty performed 7 days after EMT-RCECs injection significantly decreased due to recovered pump function. Corneal thickness 28 days after PKP in EMT-RCECs rabbits was significantly less than untreated EMT-RCECs animals (p = 1.6 × 10^−4^) (***p < 0.001). (**B**) Anterior segment photographs of rabbit corneas 7 days after RCECs transplantation, and 1, 7, 21, 28 days after penetrating keratoplasty. Eyes transplanted with EMT-RCECs showed severe edema (upper panel), while eyes after penetrating keratoplasty (PKP) recovered transparency from the day after surgery (lower panel). (**C**) IOP was similar in both groups indicating that the difference in corneal thickness was not due to difference in IOP. (EMT-RCECs: black circles *n* = 3), (PKP group: gray squares *n* = 3), *p < 0.05, **p < 0.01, ***p < 0.001, Student’s *t* test. IOP: intraocular pressure, NS: not significant.

Penetrating keratoplasty performed 7 days after injecting EMT-RCECs showed significant decrease in corneal thickness. Corneal thickness at 28 days in the PKP-treated EMT-RCECs group was lower than the untreated EMT-RCECs group (p = 1.6 × 10^−4^) (Fig. 6A). Anterior segment photographs of rabbit cornea 7 days after EMT-RCECs transplantation showed severe corneal edema. Eyes after penetrating keratoplasty (PKP) recovered transparency from the following day after surgery, and maintained corneal thickness and transparency (Fig. 6B). Intraocular pressure (IOP) was similar in both groups indicating that the difference in corneal thickness was not due to differences in IOP (Fig. 6C). These findings were consistent with what is observed in a clinical setting, where PKP is performed for human bullous keratopathy.

## Discussion

One of the mechanism of corneal endothelial dysfunction has been explained by endothelial-mesenchymal transformation (EMT)^29,33–35^. After damage to the cornea, neutrophils infiltrate the corneal endothelium and produce IL-1β^33^. Interleukin-1β subsequently activates phosphatidylinositol (PI) 3-kinase, which induces synthesis of FGF-2^34^. Fibroblast growth factor-2 triggers EMT, and forms the retrocorneal fibrous membrane characteristic of end-stage corneal endothelial disease. In this study, we developed a new rabbit corneal endothelial dysfunction model using EMT-RCECs. As far as we know, this is the first report of a suitable functional model for mimicking chronic corneal dysfunction such as Fuchs corneal endothelial dystrophy (FCED) in humans.

FCED, the most common corneal dystrophy, specifically manifests in the endothelial cell layer. The characteristic finding in FCED is the accumulation of guttae, excrescences of the Descemet’s membrane in and under the corneal endothelium. Guttae begin centrally and slowly accumulate over time, leading to endothelial cell degeneration due to increased cellular apoptosis and eventually resulting in corneal decompensation^36^. The underlying mechanisms for pathologic accumulation of extracelluar matrix in FCED are still not known. However, EMT has been suggested to be involved since TGFBI, a TGF-β-induced extracellular matrix protein, is increased in guttae of FCED corneas^37^. Multiple EMT-related gene are altered in FCED^38^, and as well as increased cytokines levels in the aqueous humor^39^. EMT-RCECs resemble chronic FECD endothelial cells in terms of decreased expression of functional corneal endothelial markers such as tight junctions formed by zonular occludens-1 (ZO-1) and Na, K-ATPase pump function. Furthermore, our model is functionally different from previous acute corneal endothelial injury models that spontaneously heal^6–18^.

Fibroblastic cells characterized by the expression of α-SMA and vimentin contribute to scarring of wounds following injury. Vimentin is expressed by most mesenchymal cells, however, the expression of α-SMA is restricted to the smooth muscle cells and a subset of fibroblastic cells designated as myofibroblasts^26,40^. Unlike humans, rabbit corneal endothelial wounds are known to spontaneously heal. In this study, α-SMA was observed spanning from corneal endothelium to corneal stroma in the EMT-RCECs group. This suggested myofibroblasts in corneal stroma during wound healing contribute to scarring of wound contraction. Interestingly, α-SMA was upregulated in the Scrape-group as well. It is possible that mechanical scraping of corneal endothelial cells without stripping the Descemet’s membrane may increase the myofibroblast phenotype in the deep corneal stroma. It is worth noting that transplanted Fresh-RCECs contributed to prevent such changes in the corneal stroma. It has also been reported that mechanical corneal endothelial injury in rabbits triggers apoptosis of posterior stromal cells in the short term^41^. Further studies are required elucidate how cell apoptosis and fibroblastic cells affects wound healing in the long term.

Levels of inflammatory cytokines in the aqueous humor are elevated during various pathologic processes such as uveitis^42^, post–cataract surgery^43^, glaucoma^44^, bullous keratopathy and low endothelial cell density^45^. We were concerned that cells cultured by TGFβ and FGF may cause various complications after transplantation into the anterior chamber. However, there was no increase in intraocular pressure and iris damage, and corneal edema outside the 8 mm zone was not observed for up to 6 weeks. Moreover, we could treat this rabbit corneal dysfunction model by PKP, which is one of the most common treatments of bullous keratopathy in humans. A clear cornea was observed following surgery, which is similar with the clinical course of PKP performed in human bullous keratopathy.

Our animal model still has limitations. First, mechanical scraping of corneal endothelium without damaging Descemet’s membrane may require a learning curve. Second, general anesthesia is required during the experiment, which may cause unintentional death of experimental animals. Third, rabbit corneal endothelial cells have different regeneration capacity compared with humans. However, despite the regeneration ability, our model showed sustained corneal endothelial dysfunction for 6 weeks. It is possible that a similar model can be used in other animal species such as mice and primates. However, rabbits are probably ideal due to the low cost and similar anatomical features with humans such as corneal diameter and thickness^46^, corneal endothelial density, and decrease in corneal diameter with age^47,48^. The mechanism of how transplanted EMT-RCECs in our model causes bullous keratopathy still needs clarification. Corneal edema may also be the result of epithelial dysfunction in addition to endothelial damage^49^. Our model is created by mechanical injury, and corneal edema is followed by epithelial bullae and subepithelial neovascularization. However, the rabbit does not have the Bowman’s layer, therefore, while our model is similar to human disease, it may not completely the same in the long term.

In summary, injection of EMT-induced RCECs within an 8-mm diameter zone of the endothelium can effectively induce bullous keratopathy in rabbits. Transplanted EMT-RCECs maintained bullous keratopathy for up to 42 days, which is much longer than acute injury models. Furthermore, the long-standing edema could be effectively treated by penetrating keratoplasty suggesting that this model is a promising tool for testing new therapies against corneal endothelial dysfunction.

## Methods

### Animal experiment approval

In all experiments, animals were housed and treated in accordance with The Association for Research in Vision and Ophthalmology Statement for the Use of Animals in Ophthalmic and Vision Research. The rabbit experiments were performed at Tokyo Dental College Ichikawa General Hospital (Ichikawa, Japan) according to the protocol approved by that university’s Animal Care and Use Committee (approval No. 297607).

### Fresh rabbit corneal endothelial cells

Eyes of Japanese white rabbits (Cat. No.J404 Funakoshi Corporation, Tokyo, Japan http://www.funakoshi.co.jp) were purchased. After enucleating the lens from the rabbit eye, the corneal endothelium, along with the Descemet’s membrane, was mechanically isolated from the cornea, removing as much tissue as possible. The isolated corneal endothelial tissue was incubated with Accutase (Cat. No. 1967954, Nacalai tesque) at 37 °C, in a humidified atmosphere of 5% CO_2_ on 35 mm dish for 30 min. After gently pipetting, the suspension was poured into a conical tube (TPP, Techno Plastic Products AG, Trasadingen, Switzerland, www.tpp.ch) through a 70 µm filter (Cat. No.352350, Falcon, Corning, NY, http://www.corning.com) to obtain single cell suspensions. Dissociated cells were centrifuged at 400 g 10 min and handled with DMEM/F12 (Cat. No. 08460-95, Nacalai tesque, Kyoto, Japan, https://www.nacalai.co.jp) supplemented with 10% fetal bovine serum (Sigma-Aldrich, St. Louis, MO, http://www.sigmaaldrich.com), penicillin (50 U/mL; Sigma-Aldrich) and streptomycin (50 μg/mL; Sigma-Aldrich). The freshly isolated RCECs were labeled with the fluorescent tracker, PKH26 (Cat. No. PKH26GL, Sigma-Aldrich) to observe cell localization after transplantation.

### EMT Cell culture

The isolated rabbit corneal endothelial cells, as noted above, were incubated at 37 °C, in a humidified atmosphere of 5% CO_2_ on 35 mm dish in the specific endothelial-mesenchymal transformation medium (SEMTM). The SEMTM was based on DMEM/F12 (Nacalai tesque) supplemented with 10% fetal bovine serum (Sigma-Aldrich), 1 ng/mL transforming growth factor (TGF) β1 (Cat. No. 240-GMP-010, R&D systems, Inc. Minneapolis, MN. USA. https://www.rndsystems.com) penicillin (100 U/mL; Sigma-Aldrich), streptomycin (100 μg/mL; Sigma-Aldrich), 1% Insulin, Transferrin, Selenium Solution (ITS-G) (Cat.No.I3146, Sigma-Aldrich), 20 ng/mL of basic fibroblast growth factor (Cat. No.AF-100-18B, PeproTech. Rocky Hill, NJ, United States. https://www.peprotech.com), 2 mM L-Ala, L-Glutamain (Cat. No.04260-64, Nacalai tesque). The SEMTM was changed every 2 days until the cells reached confluence. The harvested cells were incubated in 2 ml Accutase (Nacalai tesque) on 35 mm dish for 20 min at 37 °C after washing 3 times with phosphate-buffered saline (PBS) for passage. The suspension in 35 mm dish was poured into a conical tube (Techno Plastic Products) through a 70 µm filter (Falcon, Corning) to obtain single cell suspensions. Dissociated cells were centrifuged at 400 *g* 10 min and suspended 5.0 × 10^5^ cells in 25 cm^2^ flask with SEMTM. The harvested cells were passaged until passage 5, at which point cells were used for experimental analyses.

### Immunocytochemistry

Immunocytochemical analyses of cultured cells and fresh cornea samples were performed as described previously^50^. In brief, the cells were fixed at room temperature for 10 min in 4% formaldehyde in PBS. After 3-time PBS washes, the specimens were incubated for 30 min in Morphosave (Cat.No.250-010, Ventana Medical Systems, Inc., Tucson, Arizona, http://www.ventana.com). After 2-time PBS washes, the specimens were incubated for 30 min in 10% normal donkey serum to block nonspecific binding. This was followed by overnight incubation at 4 °C with 1: 200-diluted mouse anti-Tight junction protein 1 (ZO-1) monoclonal antibody (Cat. No. 33-910-0, Thermo Fisher Scientific, Waltham, MA, https://www.thermofisher.com) or 1: 200-diluted mouse Na, K-ATPase α1 subunit antibody (Cat.No.NB300-146, Novus Biologicals, Littleton, CO, http://www.novusbio.com) or 1: 200-diluted mouse anti-actin, α-smooth muscle-Cy3™ antibody (Cat.No.C6198, Sigma-Aldrich) or 1: 200-diluted Alexa Flour 488-Phalloidin (Cat. No.A12379, Invitrogen, Thermo Fisher Scientific, Massachusetts, USA. www.thermofisher.com) and 3-time washes in PBS. The samples were then incubated for 2 hours in a 1: 200 dilution of Cy3-conjugated donkey anti-mouse IgG antibody (Cat.No.715-165-151, Jackson ImmunoResearch Laboratories, West Grove, PA, http://www.jacksonimmuno.com) or Alexa Flour 488 conjugated donkey anti-mouse IgG antibody (Cat.No.A21202, life technologies) and again washed 3 times in the dark. Finally, the samples were mounted on dishes with an anti-fading mounting medium containing 4′,6-diamidino-2-phenylindole ([DAPI] 1 mg/ml, Cat.No.D523 Dojindo Laboratories, Kumamoto, Japan, http://www.dojindo.com). Images were obtained by fluorescent microscopy Axio imager (Carl Zeiss, Inc., Weimar, Germany, http://www.zeiss.com).

### RT-PCR analysis and quantitative RT-PCR analysis

Real-time RT-PCR and quantitative RT-PCR were performed as described previously^50^. Total RNA was purified with the use of RNeasy kit (Qiagen, Hilden, Germany, http://www.qiagen.com) and cDNA was synthesized from total RNA using the Rever Tra Ace cDNA Synthesis Kit (TOYOBO Co., Ltd., Osaka, Japan, http://www.toyobo-global.com), according to the manufacturer’s protocol. Polymerase chain reaction (PCR) was performed using Verti Thermal Cycler (Applied Biosystems, Thermo Fisher Scientific Inc.). Quantitative real time PCR with the Thunderbird SYBR qPCR Mix (TOYOBO) was performed using the Step One real-time PCR system (Applied Biosystems) in duplicate or triplicate. The primer sequences used are as follows^51^: *α-Sma*, 5′-TCTGTAAGGCCGGCTTTGC-3′ and 5′-TGTCCCATTCCCACCATCA-3′; GAPDH, 5′-ACAGTCGCCGCATCTTCTT-3′ and 5′-CTTGATTTTGGAGGGATCTCGC-3′.

### Western blot analysis

Western blot analyses were performed as described previously^50,52^. Fresh RCECs and EMT-RCECs were washed with PBS twice, and dissolved in a lysis buffer (M-PER; Thermo Fisher Scientific Inc.) with a protein inhibitor cocktail (Thermo Fisher Scientific Inc.). Western blot analysis was performed by using a standard protocol with primary antibodies for β-actin (Cat. No. ab16039, Abcam, Cambridge, U.K., http://www.abcam.com), Atp1a1 (Na-K-ATPase α-subunit) (Cat. No. NB300-146, Novus Biologicals, Littleton, Colorado, USA. http://www.novusbio.com), and Vimentin (Cat. No. sc-7557, Santa Cruz Biotechnology, Dallas, Texas, http://www.scbt.com). Chemiluminescence images were analyzed using a Charge-Coupled Device camera system (ImageQuant LAS 4000; GE Healthcare, Little Chalfont, U.K., http://www.gehealthcare.com).

### Rabbit corneal endothelial dysfunction model by injection of RCECs

Rabbit corneal endothelial dysfunction was created by modifying a previous report^14^. Japanese white rabbits (female, 2.5 kg body weight; Shiraishi Experimental Animal Breeding Farm) were anesthetized intravenously with a mixture of medetomidine hydrochloricde (0.5 mg/kg; Domitor; Meiji Seika Pharma Co., Ltd, Tokyo, Japan. http://www.meiji-seika-pharma.co.jp), midazolam hydrochlride (2.0 mg/kg; Dormicaum; Astellas Pharma, Tokyo, Japan https://www.astellas.com), and butorphanol (0.5 mg/kg; Vetorphale; Meiji Seika Pharma Co., Ltd.). Transplantation was performed on the left eye only of each animal and was performed in an animal surgery room. As preoperative treatment, Mydrin-P™ (Tropicamide Phenylephrine hydrochloride, Santen Pharmaceutical Co., Ltd, Osaka, Japan, https://www.santen.co.jp) eye drop was instilled 10 minutes before surgery. Marking was done by placing a blue pigment with Devon™ Surgical Skin Markers (Cat. No.31145942, Covidien, Medtronic plc, Dublin, Ireland. http://www.medtronic.com) on the blade of an 8 mm disposable biopsy punch (Cat. No.BP-80F, Kai medical, Tokyo, Japan. http://www.kai-group.com) and placing it vertically on the corneal epithelium. Next, the anterior chamber was punctured with a 22.5 ° straight knife (Cat. No.MST22, MANI, Tochigi, Japan. http://www.mani.co.jp) from the corneal limbus. To maintain space in the anterior chamber, OPEGAN Hi™ (Purified Sodium Hyaluronate, Santen Pharmaceutical Co., Ltd.) was injected through the puncture with a 27 G blunt needle. The corneal endothelium was mechanically scraped with a 20-gauge silicone needle (soft tapered needle; Inami, Tokyo, Japan. http://inami.co.jp) from the corneal endothelial surface within the 8 mm mark. The scraped area was confirmed by 0.04% trypan blue staining during surgery. This scraping technique and trypan blue staining were performed as described previously^14,23^. Then, the anterior chamber was irrigated with physiological saline using the Aspiration/Irrigation Unit SIMCOE™ Original Model tube (Cat. No.M-104, Inami, Tokyo, Japan). After a single suturing of 10-0 nylon, 160 µl of cell suspension was injected into the anterior chamber with a 27 gauge needle. Injected cells include cultivated RCECs and Fresh RCECs. A total of 3.0 × 10^5^ cells were suspended in 160 μl SHELLGAN™ (Purified Sodium Hyaluronate and Chondroitin Sulfate Sodium, Santen Pharmaceutical Co., Ltd, Osaka, Japan.) during injection. Endothelial scraping alone with no cell injection served as control (Scrape-group). The cornea was gently tapped to homogenize cell distribution in the anterior chamber. The operated eye was kept in a face-down position for 3 hours under general anesthesia.

Antibiotic (0.3% ofloxacin) and steroid (0.1% betamethasone) eye drops were applied topically 2 times a day. Following surgery, rabbits were carefully observed by slit-lamp microscopy, and serial photographs were obtained. Central corneal thickness was measured with an ultrasound pachymeter (Tomey, Aichi, Japan. https://www.tomey.co.jp), and intraocular pressure (IOP) was measured by Accupen (White Medical, Tokyo, Japan. http://www.whitemedical.co.jp) every 1–2 days for the first week, and then once every 2–3 days for the following 2 weeks and once every week for the following 6 weeks. Central corneal thickness measurement and IOP measurement were performed by 2 different investigators who were masked to the details of transplantation in each group. 12 eyes from 12 individual rabbits were used for each experimental group. Finally, 6 weeks after surgery, rabbits were sacrificed using the above-described anesthesia, 20 mg/kg Thiopental sodium (Ravonal, Mitsubishi Tanabe Pharma Co., Osaka, Japan. http://www.mt-pharma.co.jp), and 90 ml/kg 1 N potassium chloride (Wako, Pure Chemical Industries, Tokyo, Japan).

### Measurement of cell diameter and density

The size of corneal endothelial wounds and expansion of injected cells were measured and counted manually in randomly selected fields (n = 25), and cell density measured was measured within a square with 50 μm on one side. Images were captured at 200 x magnification for each section using a fluorescent microscopy Axio imager (Carl Zeiss, Inc.) and repeated three times under independent conditions.

### Histological examination of rabbit eyes after RCECs injection

After rabbits were sacrificed, the operated eyes were enucleated, and the cornea of each operated eye was cut, paraffin-embedded, and mounted on dishes or glass slides with an anti-fading mounting medium. The enucleated eyes were excised and fixed in 4% paraformaldehyde for 18 h. The corneal paraffin sections were used for Hematoxylin and Eosin (HE) staining, α-smooth muscle clone 1A4 antibody (Cat. No. A2547, Sigma-Aldrich, St. Louis, MO, http://www.sigmaaldrich.com) staining, vimentin (Cat. No. M0725; DAKO) staining and laminin (Cat. No. Z0097; DAKO) staining. Immunohistochemistry (IHC) staining was done automatically by BOND-MAX™ (IHC staining system, Leica Microsystems GmbH, Leica, Wetzlar, Germany, https://leica-camera.com/). The corneal images were obtained by use of a microscope BZ-9000™ (Keyence, Osaka City, Osaka, Japan, https://www.keyence.co.jp).

### Penetrating keratoplasty (PKP)

Penetrating keratoplasty (PKP) study performed by modifying a previous report^50^.

Japanese white rabbits (female, 2.5 kg body weight; Shiraishi Experimental Animal Breeding Farm) were anesthetized intravenously with a mixture of medetomidine hydrochloricde (0.5 mg/kg; Domitor; Meiji Seika Pharma Co., Ltd.), midazolam hydrochlride (2.0 mg/kg; Dormicaum; Astellas Pharma.), and butorphanol (0.5 mg/kg; Vetorphale; Meiji Seika Pharma Co., Ltd.). Transplantation was performed in an animal surgery room on the left eye only of each animal. As preoperative treatment, Mydrin-P™ (Tropicamide Phenylephrine hydrochloride, Santen Pharmaceutical Co., Ltd.) eye drop was instilled 10 minutes before surgery. Corneal buttons were prepared from donor corneas purchased from Funakoshi Co., Ltd using an 8.0-mm Barron donor cornea punch (Barron Precision Instruments, Grand Blanc, MI, USA. http://www.bpic.com). The recipient cornea was cut using a 7.5-mm Hassburg-Barron Vacuum Trephine (Cat. No. 21-8275, Barron Precision Instruments), and the corneal buttons were then sutured on the graft bed with 16 interrupted sutures (10–0 nylon). Three rabbits were transplanted with fresh corneas 7 days after EMT-RCECs injection. Antibiotics (0.3% ofloxacin) and steroids (0.1% betamethasone) were applied topically 2 times a day. After transplantation, eyes were carefully observed by slit-lamp microscopy, and serial photographs were obtained. Central corneal thickness was measured with an ultrasound pachymeter (Tomey), and intraocular pressure (IOP) was measured by Accupen (Accutome, Inc.) every 1–2 days for the first week, and then once every 2–3 days for the following 2 weeks, and once every week for the following 4 weeks. Another 3 rabbits from the EMT-RCECs group were followed without surgery as control. Finally, 28 days after surgery, rabbits were sacrificed (above-described anesthesia, 20 mg/kg Thiopental sodium (Ravonal, Tanabe Seiyaku Co., Osaka, Japan.), and 90 ml/kg 1 N potassium chloride (Wako, Pure Chemical Industries).

### Statistical analysis

Data are presented as mean ± SD and were compared by Student’s *t*-test or multiple *t*-test with Bonferroni correction after ANOVA using the Excel 2007 software (Microsoft corporation, Redmond, WA, USA, http://www.microsoft.com). *P* value of <0.05 was considered statistically significant.

## Electronic supplementary material

Supplementary Information
