# Supplementary material for: A Rabbit Corneal Endothelial Dysfunction Model Using Endothelial-Mesenchymal Transformed Cells
Source: Sci Rep. 2018 Nov 15;8:16868. doi: 10.1038/s41598-018-35110-2 (PMC6237874; doi:10.1038/s41598-018-35110-2)

Title;
A Rabbit Corneal Endothelial Dysfunction Model Using Endothelial-Mesenchymal Transformed Cells

Running title;
Rabbit Corneal Endothelial Dysfunction Model

Names of authors;
Kazuya Yamashita[1], Shin Hatou[1], Emi Inagaki[1], Kazunari Higa[2], Kazuo Tsubota[1], Shigeto Shimmura[1*]

Names of institution;
[1] Department of Ophthalmology, Keio University School of Medicine, Tokyo, Japan
[2] Department of Ophthalmology, Tokyo Dental College Ichikawa General Hospital, Ichikawa, Japan

*Corresponding information; E-mail: shige@z8.keio.jp

Supplementary Figure 1

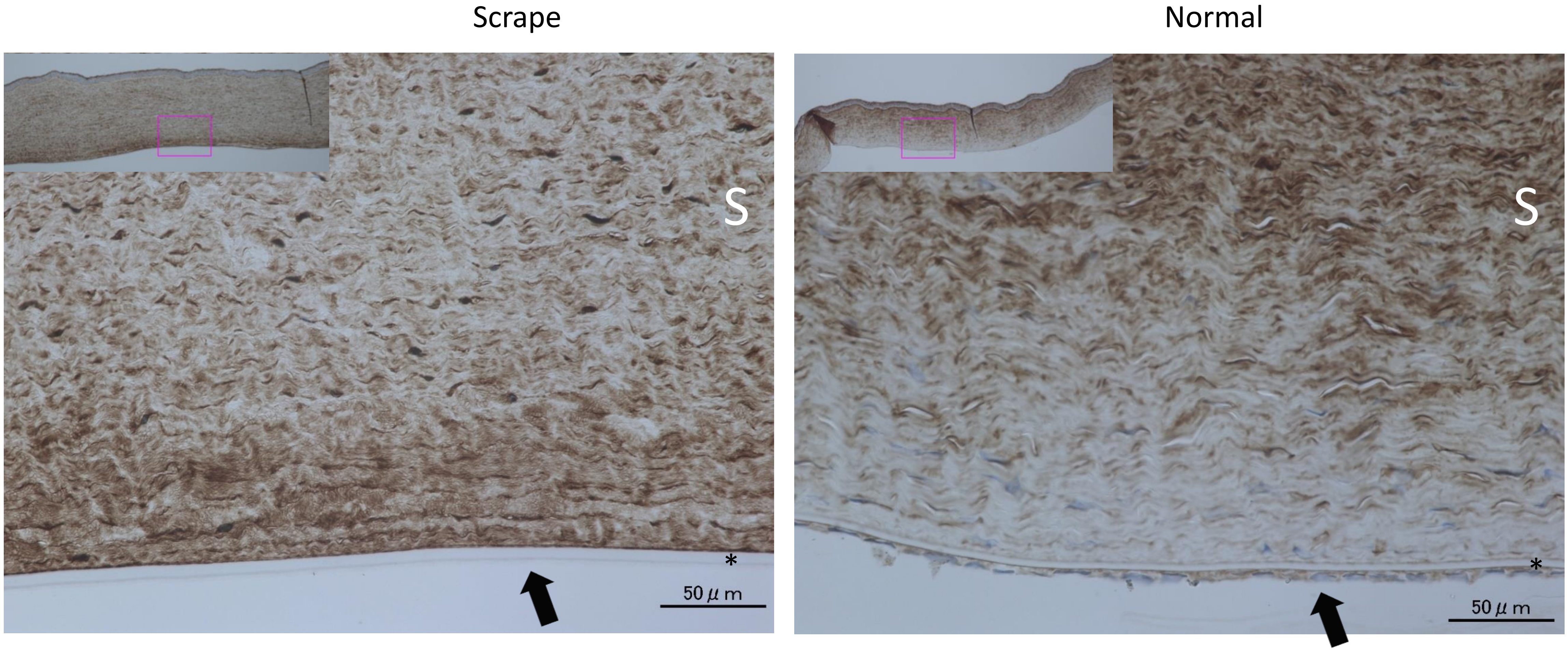

Supplementary Figure 1. Immunohistochemistry for laminin in scraped and intact corneas.
Laminin staining was observed along the Descemet’s membrane surface (arrows), and within the corneal stroma (S). Supplementary Figure 1 showed that the laminin layer is intact on the Descemet’s membrane surface following cell scraping. (Abbreviation, S : Corneal stroma, * : Descemet’s membrane)

Supplementary Figure 2

Original gel images used in Figure 1B.

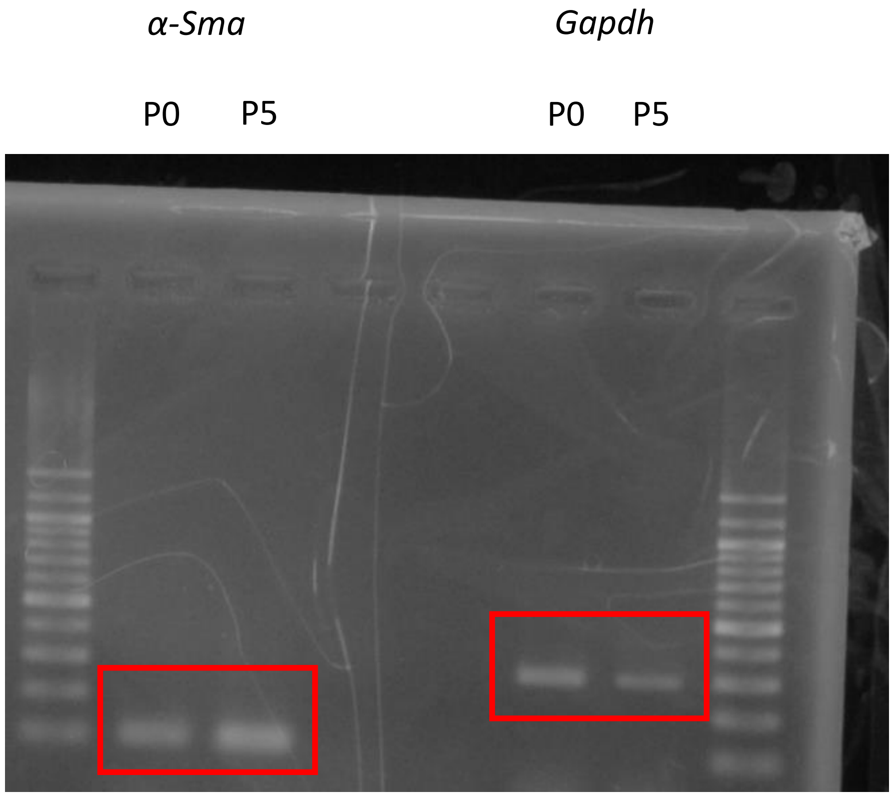

Supplementary Figure 3

Original gel and blots used in Figure 1C.

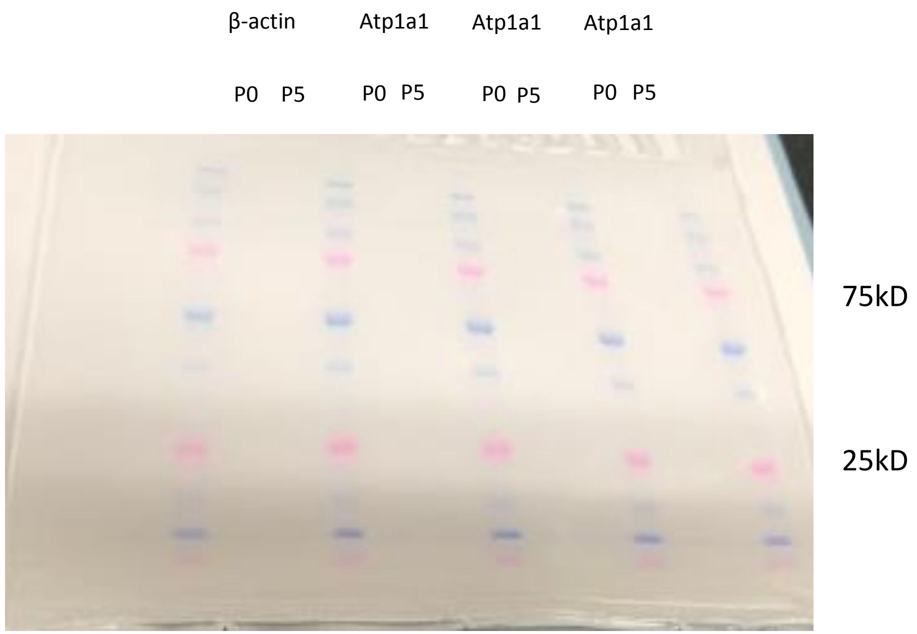

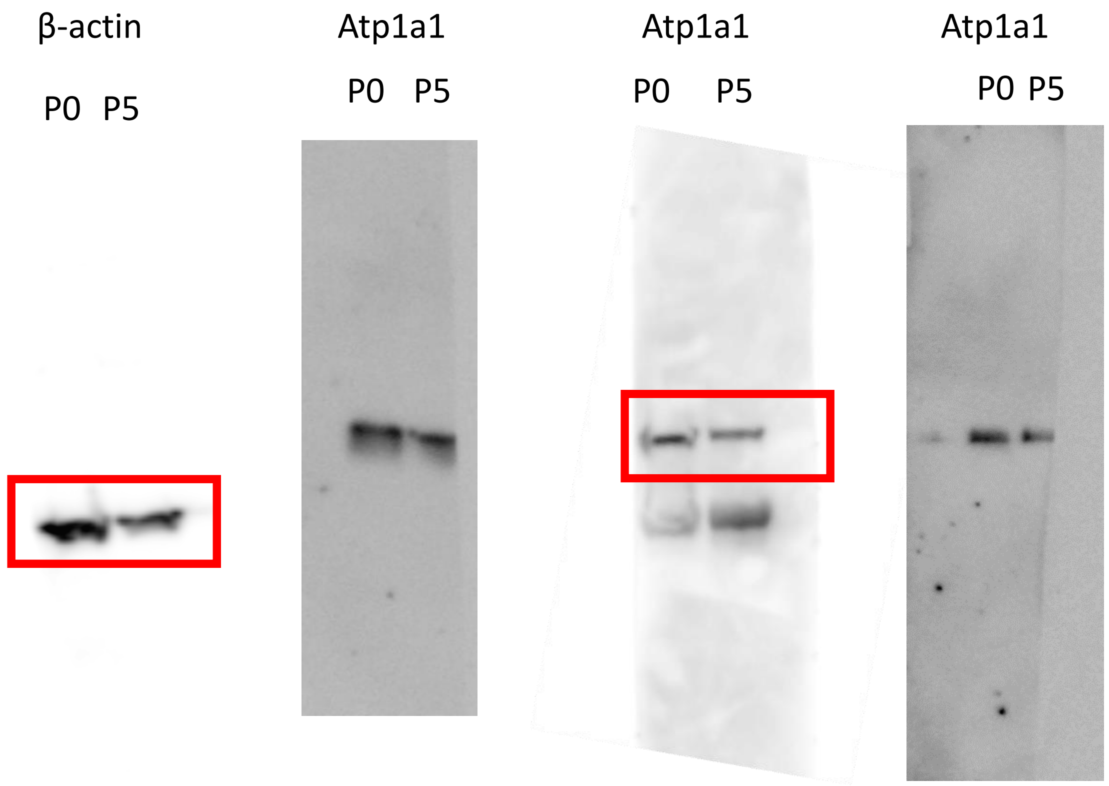

Supplementary Figure 4

Original gel and blots used in Figure 1C.

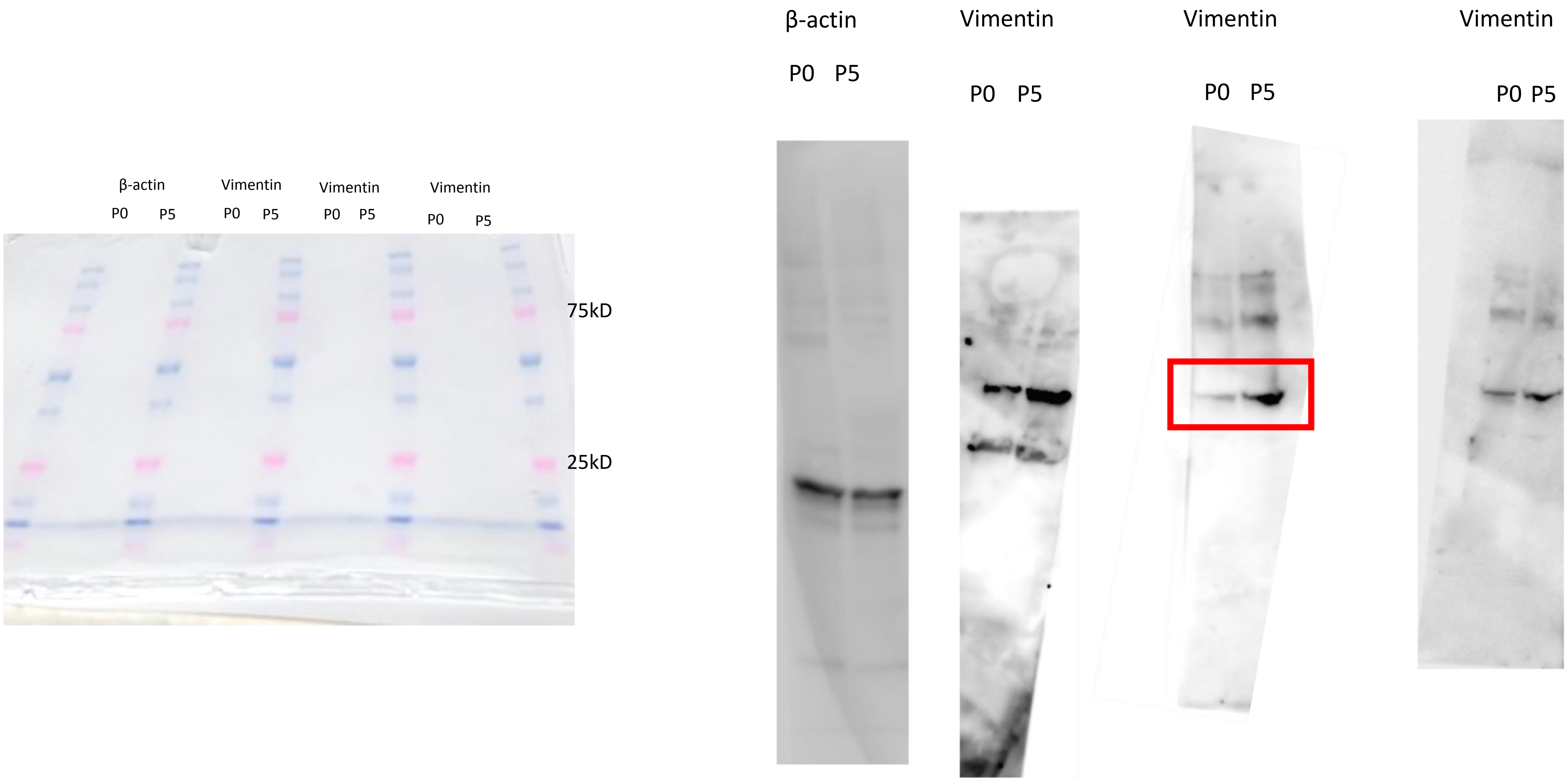

Supplement: Supplementary file 1 — Supplementary Information [file 41598_2018_35110_MOESM1_ESM.pdf]
